# Improved base-calling and quality scores for 454 sequencing based on a Hurdle Poisson model

**DOI:** 10.1186/1471-2105-13-303

**Published:** 2012-11-15

**Authors:** Kristof De Beuf, Joachim De Schrijver, Olivier Thas, Wim Van Criekinge, Rafael A Irizarry, Lieven Clement

**Affiliations:** 1 Department of Mathematical Modelling, Statistics and Bioinformatics, Ghent University, Coupure Links 653, B9000 Ghent, Belgium; 2 Centre for Statistical and Survey Methodology, School of Mathematics and Applied Statistics, University of Wollongong, NSW 2522, Australia; 3 Department of Biostatistics, Johns Hopkins Bloomberg School of Public Health, 615 N. Wolfe St., Baltimore, MD, USA; 4 Department of Applied Mathematics and Computer Science, Ghent University, Krijgslaan 281-S9, B9000 Ghent, Belgium; 5 Interuniversity Institute for Biostatistics and Statistical Bioinformatics, Katholieke Universiteit Leuven and Universiteit Hasselt, Kapucijnenvoer 35, Blok D, bus 7001, B3000 Leuven, Belgium

## Abstract

**Background:**

454 pyrosequencing is a commonly used massively parallel DNA sequencing technology with a wide variety of application fields such as epigenetics, metagenomics and transcriptomics. A well-known problem of this platform is its sensitivity to base-calling insertion and deletion errors, particularly in the presence of long homopolymers. In addition, the base-call quality scores are not informative with respect to whether an insertion or a deletion error is more likely. Surprisingly, not much effort has been devoted to the development of improved base-calling methods and more intuitive quality scores for this platform.

**Results:**

We present HPCall, a 454 base-calling method based on a weighted Hurdle Poisson model. HPCall uses a probabilistic framework to call the homopolymer lengths in the sequence by modeling well-known 454 noise predictors. Base-calling quality is assessed based on estimated probabilities for each homopolymer length, which are easily transformed to useful quality scores.

**Conclusions:**

Using a reference data set of the *Escherichia coli* K-12 strain, we show that HPCall produces superior quality scores that are very informative towards possible insertion and deletion errors, while maintaining a base-calling accuracy that is better than the current one. Given the generality of the framework, HPCall has the potential to also adapt to other homopolymer-sensitive sequencing technologies.

## Background

A first step in the analysis of next-generation sequencing (NGS) data is the transformation of the measured intensity signals to a sequence of nucleotides. This process, referred to as base-calling, is an important task, as systematic base-calling errors may mislead downstream analysis [1], e.g. in genome assembly and sequence mapping. More accurate base-calling and more reliable base-calling quality scores result in a better distinction between sequencing errors and true polymorphisms between the base-called reads and a reference sequence. This is an essential merit in the detection of single nucleotide polymorphisms (SNPs) or sequence variants [2-4]. A myriad of applications such as the characterization of HIV mutation spectra [5], the detection of somatic mutations in cancer [6], and the identification of operational taxonomic units in metagenomics [7] have the potential to benefit from improved base-calling and more informative quality scores.

The 454 Life Sciences system is based on the sequencing-by-synthesis principle. In each flow of the sequencing process, light produced by a pyrosequencing reaction is emitted if one or more identical nucleotides are incorporated into the DNA template. The addition of each of the 4 possible nucleotide solutions A, C, G or T occurs in a fixed and known order. Hence, 454 base-calling is a matter of discerning the number of incorporated nucleotides or homopolymer length (HPL) of a known nucleotide type from the measured intensity signal in each flow [8]. Consequently, the principal sources of 454 sequencing errors are insertion and deletion errors (indels). These are more frequent in sequences containing long homopolymers [9,10], because the increase of intensity signal when more nucleotides are incoporated, attenuates at higher HPLs. This makes it harder to discriminate between subsequent homopolymer lengths, resulting in an inflation of undercalls or overcalls as the HPL increases (Additional file 1: Figure S1).

**Figure 1:**
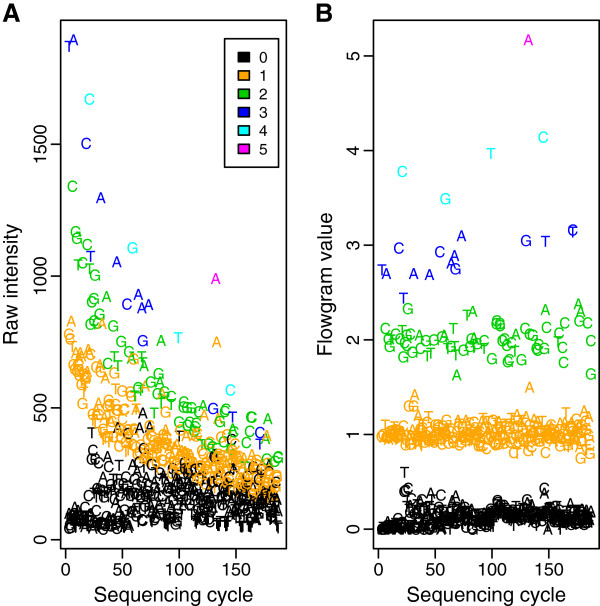
**The effect of preprocessing raw intensities.** Raw intensities and flowgram values versus cycle number for a typical read, illustrating the effect of preprocessing. The colors represent the reference HPL. The preprocessing of raw intensities to flowgram values removes much noise. However, the raw intensities still contain additional information that can be used in the base-calling.

In the default 454 sequencing pipeline the raw intensities are preprocessed to flowgram values by correcting for the major error sources. These include spatial and read-specific effects such as the abundance of long homopolymers in a read [8,11]. This preprocessing eliminates much obscuring noise, but removes some useful information as well (Figure 1). In the next step the base-calling takes place, where, roughly speaking, the flowgram values are rounded to the nearest integer. Subsequently, a quality score is assigned to each called base. Quality score calculation in current 454 base-calling is solely based on the flowgram values without considering information from the preprocessing step [11]. An alternative method for quality score assignment has been proposed that focuses on the distribution of observed flowgram values for every possible HPL [4], but it does not account for additional error sources. A common feature is that such methods are designed as a next step in the pipeline after the base-calling is finished. Hence, the base-calling uncertainties inherent to the base-calling model or algorithm are not directly utilized in the construction of the quality scores.

**Figure 2:**
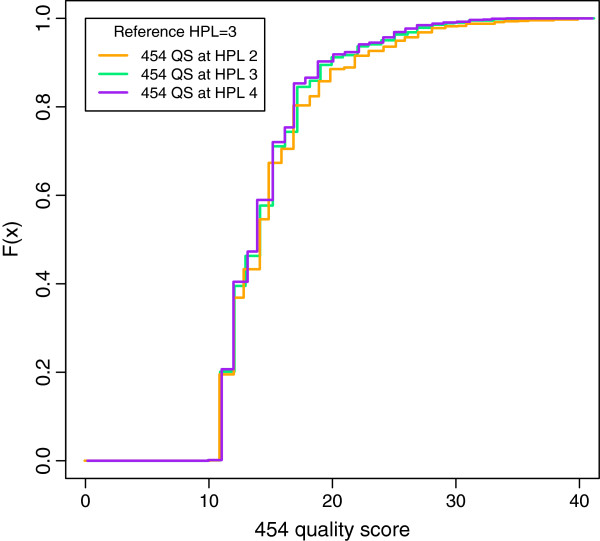
**Distribution of quality scores by 454 native base-caller at HPLref 3 overcall.** The empirical cumulative distribution functions of 454 quality scores assigned to bases associated with HPL 2, 3 and 4 in case of an overcall for sequences with reference HPLref 3. The distributions are nearly identical, implying that no insight is provided by the 454 quality scores about whether an undercall or an overcall is more likely, given that a base-calling error was made.

A second shortcoming of the current base-callers is that they only produce quality scores in the *Phred* format [12]. *Phred* scores can be interpreted in terms of the probability that the called base is not an overcall. Although well-known and widely used, they provide a measure for the quality of the base-call, but they lack additional information on whether there is an undercall or an overcall, and on how likely it is for other HPLs to be the correct call, instead of the HPL being called. This feature is particularly essential in 454 pyrosequencing. An example of overcalls of reference sequences with HPLref 3 shows that the distributions of 454 quality scores are nearly identical for the called bases associated with position 2, 3 and 4 in the homopolymer run (HPL 2, 3 and 4) (Figure 2). Hence, these quality scores do not give any insight into whether it is more likely to have an undercall or an overcall, given that a base-calling error was made. This information would, however, be very useful in downstream tasks such as sequence alignment and sequence variant calling. Quite some methods have recently been proposed that consider quality scores to increase accuracies in these downstream analyses, e.g. [13-15]. By adding more detailed information about the probabilities of having an undercall or overcall, these methods could be improved even further.

For these reasons we have developed HPCall, a general probabilistic framework that seamlessly integrates the base-calling with more informative quality score assignment. HPCall is based on the classification of the calls in groups representing the possible HPLs. To this end, a statistical model for count data is used that predicts the HPL in each sequencing cycle as a function of a number of explanatory variables. A particular property of the 454 pyrosequencing process is the high abundance of background intensities (HPL 0). Whenever a nucleotide flow does not match the nucleotide at the interrogated position of the DNA template, no nucleotide is incorporated and no sequencing reaction takes place. The resulting light intensity mainly reflects background optical noise. Consequently, there are more zero counts than expected for a Poisson distribution. Hurdle Poisson regression models [16] are one way to deal with these excess zeros.

For each possible HPL, the model produces an estimated probability that this HPL is truly present in the DNA sequence at the interrogated position. The called HPL is then the HPL with the largest estimated probability. The calculation of these probabilities allows the simultaneous construction of quality scores. These scores directly reflect the base-calling’s uncertainties and provide information about potential undercall or overcall errors. In the model we combine information of flowgrams and earlier-stage raw intensities. By including the raw intensities we employ the additional information otherwise removed by the preprocessing (Figure 1), both for the base-calling and for the calculation of the quality scores. However, they are not strictly necessary for the method to provide valid results.

## Methods

### Sequencing data

To assess the base-calling accuracies, DNA of the reference K-12 strain MG1655 of the bacterium *Escherichia coli* was sequenced at the NXTGNT sequencing center, using shotgun sequencing with Titanium reagents. Results are presented for a random subset of 15000 out of the 635979 produced reads. The reads in the standard 454 pipeline were produced using software version 2.3.

### Base-calling pipeline

Before running the Hurdle Poisson base-calling model a preliminary data preparation step is performed in HPCall. In this step several raw data files are merged to create a data set that can be used for calibration of the model, if needed, and for subsequent base-calling. After the base-calling three output files are created: (a) the base-called reads, (b) the associated *Phred*-like quality scores, and (c) a file with the base-calling probabilities by HPL. A visualization of the base-calling pipeline, including a more detailed description, can be found in the Additional file (Additional file 2: Figure S2).

### Weighted Hurdle Poisson model

#### Model specification

Let *N*_*bc*_ be the number of nucleotides *b* that are incorporated in cycle *c*, with *b* ∈ *B* = {*ACTG*} and *c* = 1,…,*L*, where *L* represents the total number of cycles in the sequencing experiment. Note that one cycle consists of 4 flows of nucleotide solutions added in fixed order. The base-calling problem is treated as a classification problem, where *N*_*bc*_ is the class indicator. Based on the observed input information on the raw intensities and flowgram values, the flows are assigned to one of these classes. If there are only two possible classes, this is often done by logistic regression. Here, we use Poisson regression, because multiple HPLs have to be classified. Furthermore, these models also allow for extrapolation to larger HPLs. To model the excess zeros in the data we consider Hurdle Poisson models. They are mixture models with a binomial component that distinguishes between zero counts and positive counts, and a zero-truncated Poisson component which models the positive counts, conditional on having a non-zero count. Because 454 data show considerable underdispersion after truncation, i.e. the variance is smaller than the mean, a weighted Poisson component [17] is adopted in the model to cope with this. The following Hurdle Poisson model is considered:

(1) $$\text{Pr}\{N_{\text{bc}}=n_{\text{bc}}\}=\left\{\begin{array}{ll} 1-\Pi_{\text{bc}} & \text{if}\;n_{\text{bc}}=0\\ \Pi_{\text{bc}}\;f_{\text{ZTWP}}(n_{\text{bc}};\lambda_{\text{bc}},\theta ) & \text{if}\;n_{\text{bc}}=1,2,3,\ldots \end{array}\right)$$

where the probability *Pr*{*N*_*bc *_=* n*_*bc*_} is conditional on the observed raw intensities and flowgram values, and *f*_ZTWP_ is the density of a zero-truncated weighted Poisson distribution which is given by

(2) $$f_{\text{ZTWP}}=\frac{f_{\mathrm{WP}}(n_{\text{bc}};\lambda_{\text{bc}},\theta )}{1-f_{\mathrm{WP}}(0;\lambda_{\text{bc}},\theta )}\;\text{for}\;n_{\text{bc}}=1,2,3,\ldots ,$$

with *f*_WP_ denoting the density of the weighted Poisson distribution,

(3) $$f_{\mathrm{WP}}(n_{\text{bc}};\lambda_{\text{bc}},\theta )=\frac{w_{n_{\text{bc}}}e^{-\lambda_{\text{bc}}}\lambda_{\text{bc}}^{n_{\text{bc}}}}{W_{\text{bc}}n_{\text{bc}}!}.$$

In (3), $\left\{w_{n_{\text{bc}}}\right\}$ denotes a set of weights, *λ*_*bc*_ > 0 and the normalizing constant is given by $W_{\text{bc}}=\overset{\infty }{\underset{n_{\text{bc}}=0}{\sum }}\frac{e^{-\lambda_{\text{bc}}}\lambda_{\text{bc}}^{n_{\text{bc}}}w_{n_{\text{bc}}}}{n_{\text{bc}}!}$. We use exponential weights $w_{n_{\text{bc}}}$ similar to the ones proposed in [17],

(4) $$w_{n_{\text{bc}}}=e^{-\theta {\left(\lambda_{\text{bc}}-n_{\text{bc}}\right)}^{2}}\;\text{with}\;\;\theta >0.$$

By considering a positive *θ* the underdispersion in the count data can be modeled properly.

The nucleotide- and cycle-specific parameters *Π*_*bc *_in the binomial component and *λ*_*bc *_in the Poisson component are modeled with several predictors. We allow the predictor effects to be nonlinearly associated with the HPL by considering generalized additive models (GAM) [18]. In particular,

(5) $$\text{logit}(\Pi_{\text{bc}})=\beta_{0,\text{bc}}+\overset{k}{\underset{j=1}{\sum }}f_{j}(x_{j,\text{bc}}),$$

(6) $$\text{log}(\lambda_{\text{bc}})=\gamma_{0,\text{bc}}+\overset{l}{\underset{j=1}{\sum }}g_{j}(y_{j,\text{bc}}),$$

with the *f*_*j *_and *g*_*j *_being smooth functions of the corresponding predictor variables *x*_*j*,*bc *_and *y*_*j*,*bc*_, respectively. Details about the smooth functions are provided in the Additional file 3. Note that the predictor variables in (5) and (6) can be specified separately. In this paper we propose that the following information is used in either or both of the 2 submodels: 

• flowgram values in the current flow;

• log_2_ raw intensities in the current flow with or without a read-specific normalization;

• the cumulative sum of flowgram values and log_2_ raw intensities up to the current flow; this allows for modeling a cycle-specific effect and recognizes the abundance of homopolymers in the preceding flows;

• flowgram values of 1, 4 and/or 8 flows before and/or after the current flow; this corrects for homopolymers in preceding and subsequent flows.

#### Parameter estimation or model training

The model parameters are estimated by maximum likelihood. This is done by using an iteratively reweighted least squares procedure [19] for both submodels. Efficient fitting of the model is conducted by the -package [20].

The estimation of the model parameters is based on the use of a representative training data set generated from a reference DNA sequencing experiment. The corresponding reference HPLs are the class indicators in the classification model. For the *E. coli* reference run we randomly selected 1000 reads to fit the base-calling model. The remaining 14000 reads in the subset were used for assessing the performance of the base-calling method.

### Base-calling and quality score construction

In flow *bc*, HPCall calls the HPL *n*_*bc *_for which $\hat{P}\{N_{\text{nc}}=n_{\text{bc}}|x_{j,\text{bc}},y_{j,\text{bc}}\}$ is maximal. These probabilities are obtained by plugging the estimated parameters from submodels (5) and (6) into model (1). They are also very useful quality scores because they provide a direct probabilistic interpretation to the base-calling uncertainties and give insight into potential undercall or overcall errors. Moreover, they can also be used for the construction of *Phred* scores in a similar fashion as the traditional 454 quality scores: it is a quality score that reflects the probability that the called base is not an overcall. In particular, the *Phred*-like quality score of the *k*-th called base in a homopolymer stretch (*k* > 0) is thus given by: $QS_{k,\text{overcall}}=-10\;{\text{log}}_{10}(\overset{\infty }{\underset{n_{\text{bc}}=k}{\sum }}\hat{P}\{N_{\text{bc}}=n_{\text{bc}}|x_{j,\text{bc}},y_{j,\text{bc}}\})$. Since we can obtain the probabilities for all possible HPLs, we can also calculate an alternative quality score that reflects the probability that the called base is not an undercall. This is given by $QS_{k,\text{undercall}}=-10\;{\text{log}}_{10}(\overset{k}{\underset{n_{\text{bc}}=0}{\sum }}\hat{P}\{N_{\text{bc}}=n_{\text{bc}}|x_{j,\text{bc}},y_{j,\text{bc}}\})$. Using *QS*_*k*,overcall_ and *QS*_*k*,undercall_ a new quality score is calculated: *QS*_*k*,HPCall_ = *I*_dir_ × min(*QS*_*k*,undercall_,*QS*_*k*,overcall_) with *I*_dir_ = − 1 if *QS*_*k*,undercall_ < *QS*_*k*,overcall_ and *I*_dir_ = 1 if *QS*_*k*,undercall_ > *QS*_*k*,overcall_. The sign of *QS*_*k*,HPCall_ thus indicates whether an undercall or an overcall is more likely.

### Performance evaluation

The performance of HPCall is compared with that of the native 454 base-caller and of Pyrobayes [4] based on the *E. coli* reference run. The *Phred*-like quality scores produced by the different base-callers are compared to ‘observed’ quality scores. The latter are computed by grouping all the bases with an equal quality score together, and computing for each group the proportion of overcalls. An observed quality score is calculated as *QS*_observed_ = −10 log_10_(observed overcall error rate). High HPCall quality scores are trimmed to 40, just like it is done by the native 454 base-caller. Further, the proportions of high-quality bases for the different base-callers are compared. Next, we illustrate the added value of the HPCall base-calling probabilities and the new quality scores *QS*_HPCall_.

The raw base-calling accuracy is assessed to give insight into the base- and read-level error rates. For HPCall the reproducibility of this accuracy is evaluated based on 10 random training data sets. Subsequently, an indel and SNP-analysis is conducted using variant detection software. We use both *ssaha2*[21] and *subread*[22] ( http://sourceforge.net/projects/subread/) to map the base-called reads to the reference sequence and *ssahaSNP*[23] to compute the number of sequence variants, both SNPs and indels. False positive calls are determined by comparing the base-calls to the *E. coli* K-12 strain reference genome. Finally, the computational performance for the different methods is compared.

## Results

### Quality scores and base-calling probabilities

**Figure 3:**
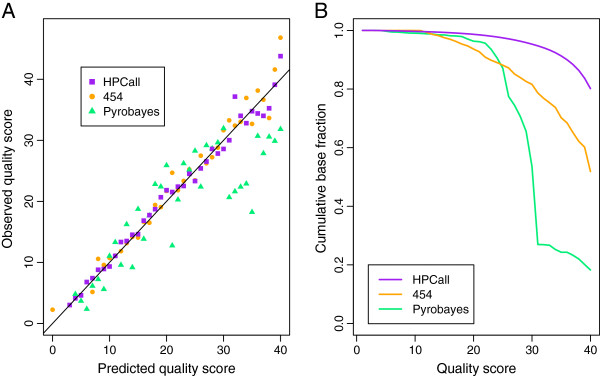
**Comparison of quality score assignment.** Comparison of quality score assignment. **(A)** Observed versus predicted quality score. Both HPCall and the native 454 base-caller have an equally accurate quality score assignment, whereas Pyrobayes performs clearly worse. **(B)** Cumulative proportion of called bases versus the assigned quality score. HPCall assigns more high quality scores than the other base-callers; the left figure suggests that this assignment is done accurately.

HPCall provides estimated probabilities that a certain HPL is present given the values of all the input variables in the model. These probabilities are thus the most direct way to quantify the base-calling uncertainty. In addition, they can also be used to compute *Phred*-like quality scores as generated by the native 454 base-caller and by Pyrobayes. The quality score assignment of the different base-callers is assessed by comparing the predicted quality score with the observed quality score for the *E. coli* data set. Both for HPCall and for the native 454 base-caller the predicted quality scores seem to reflect the observed quality quite well, and the quality score assignment seems equally good (Figure 3A). For Pyrobayes the performance is clearly worse, as predicted high quality scores overestimate the true quality of the base-calls. We also observe that HPCall generates more high quality scores than the other two base-callers (Figure 3B). As an illustration, HPCall assigns to 95% of the called bases a quality score of 30 or more, whereas this cumulative base fraction is only 82% for the native 454 base-caller and 54% for Pyrobayes.

**Figure 4:**
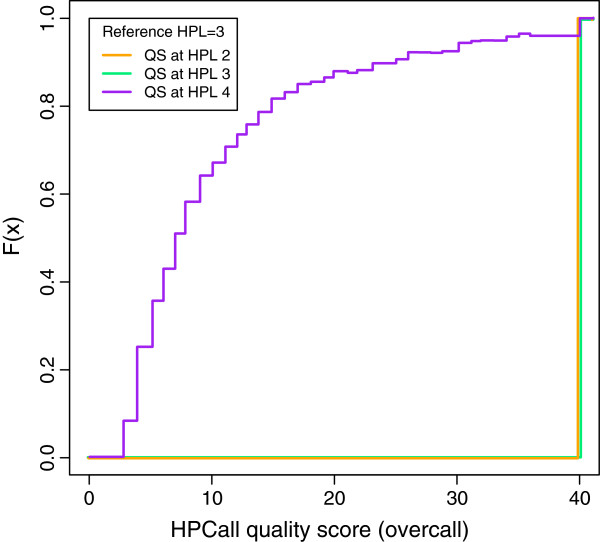
**Distribution of HPCall quality scores at HPLref3 overcall.** The empirical cumulative distribution function of HPCall quality scores *QS*_overcall_ assigned to bases associated with HPL 2, 3 and 4 for sequences with reference HPLref 3. The quality scores associated with HPL 2 and HPL 3 are generally large, whereas HPL 4 gives smaller quality scores. HPCall clearly indicates that overcalls are more likely in this situation, whereas this insight is not provided by the native 454 quality scores.

In the introduction we have argued that the 454 quality scores do not give any insight into whether it is more likely that a possible undercall or overcall was made (Figure 2). Using the same example of overcalls for reference sequences with HPLref 3, the HPCall quality scores *QS*_overcall_ clearly indicate that overcalls are more likely (Figure 4) in this situation. This can be seen from the large quality scores associated with HPL 2 and HPL 3, whereas HPL 4 gives smaller quality scores. A similar picture is seen for the undercalls of reference sequences with HPLref 3, based on *QS*_undercall_ (Additional file 4: Figure S3). Note that this plot can not be made for 454 quality scores. Plots with respect to the combined HPCall quality score *QS*_HPCall_ reveal that the sign of these quality scores provides additional information about whether an undercall or an overcall is more likely (Additional file 5: Figure S4).

**Figure 5:**
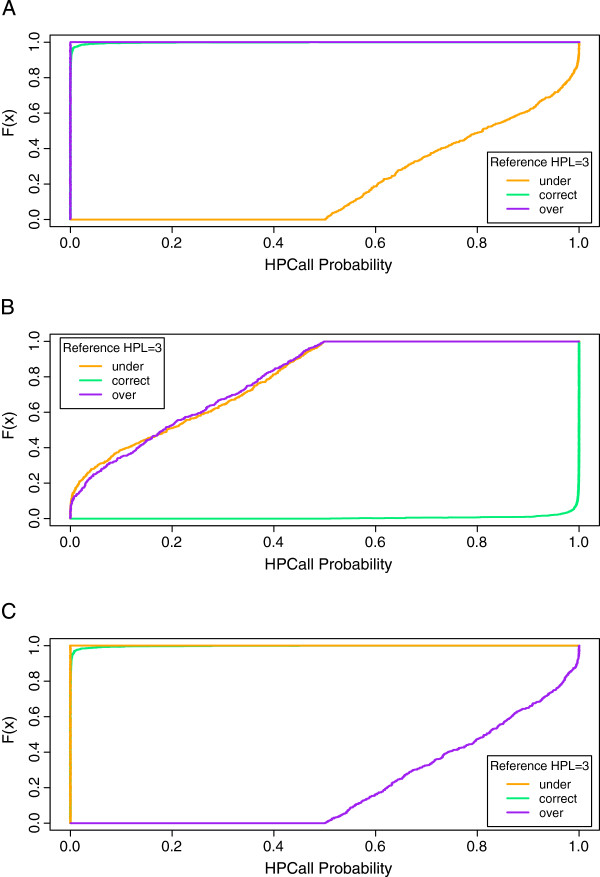
**Distribution of HPCall base-calling probabilities.** The empirical cumulative distribution functions of probabilities estimated by HPCall for sequences with reference HPLref 3. The depicted probabilities are **(A)** cumulative sum of probabilities below HPL 3, **(B)** probabilities at HPL 3, and **(C)** cumulative sum of probabilities above HPL 3. In each panel the cumulative distribution functions are plotted separately in case of an undercall, correct call or overcall. The distributions of probabilities clearly indicate larger base-calling uncertainties in case of an undercall or overcall.

As mentioned before, the *Phred*-like HPCall quality scores are based on estimated probabilities of being the correct call. Hence, these probabilities are also very useful to assess the base-calling quality. Their distribution for the example of reference sequences with HPLref 3 shows that undercalls and overcalls are associated with larger base-calling uncertainties than correct calls (Figure 5). In case of a correct call almost all probabilities at HPL 3 are very close to 1, whereas the cumulative sum of probabilities below HPL 3 in case of an undercall and above HPL 3 in case of an overcall are more evenly distributed between 0.5 and 1. In case of a miscall the estimated probability at the reference HPL very often is second largest. Moreover, the miscalled maximal probability and the probability at the reference HPL nearly always sum to a value close to 1 (Additional file 6: Figure S5).

The merit of having the base-calling probabilities at our disposal is further demonstrated by examining indels that are flagged in sequence variant detection (see also further). In the first example (Table 1) an undercall with respect to the reference sequence *AAAAA* is called by both HPCall and the native 454 base-caller. The native 454 base-caller assigns a quality score of 22 to the fourth *A* in the homopolymer sequence. This score of 22 does not indicate whether it is more likely that the fourth called *A* is a potential under- or overcall. Either way, there is no fifth quality score available to provide more information about a possible fifth *A* to be called. For HPCall we have the additional information that the estimated probability that there should be five *A*’s called is 0.17. This indicates that a miscall for this flow would almost certainly be an undercall. This is confirmed by the negative sign of *QS*_HPCall_ = −8 for this example. It is obvious that mapping algorithms that take this additional information into account will be able to more reliably map the base-called reads to the reference sequence. A very similar situation is observed in the case of an overcall (Table 2). A homopolymer stretch *AA* is considered in the reference sequence, but is called as *AAA* by both base-callers. Again, the quality score of 23 given by the native 454 base-caller for the third *A* does not give an indication of the probability of having an undercall or an overcall, given that there is a miscall. HPCall on the other hand does provide this information. Since the estimated probability of HPL 2 is 0.29, an overcall seems much more likely than an undercall. Also here this is confirmed by the positive sign of *QS*_HPCall_ = 5. Finally, an example of the special situation is considered where no base is called while there is one in the reference sequence (Table 3). Because the native 454 base-caller only produces a quality score for every called base, there is no quality score provided in this situation. Hence, there is no indication of the uncertainty of not having a call in the current flow. HPCall estimates the probability of having HPL 0 at 0.75, and of having HPL 1 at 0.25, with an associated *QS*_HPCall_ of −6, indicating that it is not unlikely that there should be one base called instead of none.

**Table 1:** Base-calling probabilities example 1: undercall

***reference sequence: AAAAA***					
***native 454: AAAA***					
	qs 2	qs 3	**qs 4**	qs 5	qs 6
*QS*_454_	28	22	**22**	-	-
***HPCall: AAAA***					
	HPL 2	HPL 3	**HPL 4**	**HPL 5**	HPL 6
$\hat{P}\{N_{\text{nc}}=n_{\text{bc}}\}$	<1E-15	7.4E-9	**0.83**	**0.17**	6.3E-11
*QS*_HPCall_	0	0	**-8**	1	0

The estimated probability for HPL 5 (0.17) and the associated *QS*_HPCall_ (-8) provided by HPCall clearly indicate the possibility of having an undercall with respect to the reference sequence, whereas this information is lacking in the quality scores of the native 454 base-caller.

**Table 2:** Base-calling probabilities example 2: overcall

***reference sequence: AA***					
***native 454: AAA***					
	qs 1	qs 2	**qs 3**	qs 4	qs 5
*QS*_454_	22	22	**23**	-	-
***HPCall: AAA***					
	HPL 1	**HPL 2**	**HPL 3**	HPL 4	HPL 5
$\hat{P}\{N_{\text{nc}}=n_{\text{bc}}\}$	1.7E-10	**0.29**	**0.71**	3E-9	<1E-15
*QS*_HPCall_	0	-1	**5**	0	0

Base-calling probabilities example 2: overcall. HPCall gives a probability for HPL 3 of 0.29 and an associated *QS*_HPCall_ of 5. This indicates that it is not unlikely that the fourth called *A* is an overcall. This information can not be extracted from the quality scores of the native 454 base-caller.

**Table 3:** Base-calling probabilities example 3: 0-1 undercall

***reference sequence: T***					
***native 454: -***					
	**qs 0**	qs 1	qs 2	qs 3	qs 4
*QS*_454_	**-**	-	-	-	-
***HPCall: -***					
	**HPL 0**	**HPL 1**	HPL 2	HPL 3	HPL 4
$\hat{P}\{N_{\text{nc}}=n_{\text{bc}}\}$	**0.75**	**0.25**	2.9E-8	<1E-15	<1E-15
*QS*_HPCall_	**-6**	1	0	0	0

Because there is no base called by the native 454 base-caller, also no quality score is provided. HPCall on the other hand generates probabilities for HPL 0 (0.75) and HPL 1 (0.25) and an associated *QS*_HPCall_ at HPL 0 of -6, indicating that an undercall is not unlikely.

### Prediction accuracy

**Figure 6:**
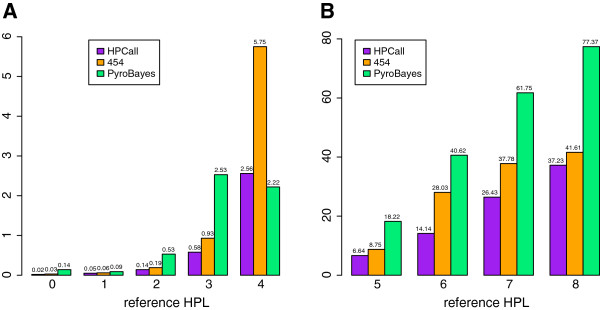
**Comparison of base-calling errors.** Comparison of the percentages of base-calling errors by HPL for the three base-calling methods. Using HPCall leads to an overall decrease of the number of base-calling errors by 35% as compared to the native 454 base-caller. The lower number of base-calling errors for HPCall is consistent throughout the complete range of reference HPLs.

The prediction accuracy of HPCall is examined for the *E. coli* data and compared with the performance of the native 454 base-caller and Pyrobayes. Based on the 14000 evaluation reads in the *E. coli* reference run, an overall decrease of 35% of the percentage of base-calling errors is observed for HPCall as compared to the native 454 base-caller (Figure 6). The lower number of base-calling errors is consistent throughout the whole range of HPLs, with peaks at HPL 4 (-55%) and HPL 6 (-50%). A plot of the absolute number of base-calling errors is shown in the Additional file (Additional file 7: Figure S6). These results are based on using information from both the raw intensities and the flowgram values. If only flowgram values are used, the prediction accuracy is slightly smaller but still larger as compared to the competing base-callers (Additional file 8: Table S1). Sensitivity analysis indicates that HPCall prediction accuracies are very stable across different training data sets (Additional file 9: Figure S7).

### Read-wise assessment and sequence variant analysis

The reads produced by HPCall are mapped to the reference sequence using *ssaha2* and *subread*. In the mapping of the HPCall reads the traditional quality scores produced by HPCall without sign information are used. The read-wise error rate is compared to the native 454 base-caller (Additional file 10: Table S2). For this data set mapping percentages of 99.47% (ssaha2) and 99.43% (subread) are obtained. 66% (ssaha2) to 69% (subread) of the reads produced by HPCall map perfectly to the reference genome of the *E. coli* K-12 strain, whereas this is only the case for 56% (ssaha2) to 60% (subread) of the reads produced by the native 454 base-caller. This evidently leads to a higher percentage of 454 reads with at least one mismatch to the reference genome as compared to reads generated by HPCall. The mapping location of the reads produced by HPCall and those produced by the native 454 base-caller differed by more than 10 bp for only 2 reads. The good performance of HPCall was confirmed by a read-wise assessment on data of a 454 amplicon resequencing experiment of the human TP53 gene. More details can be found in the Additional file 11. Sequence variants of the mapped reads are detected by the *ssahaSNP* program (Additional file 10: Table S3). A reduction of the number of sequence variants with 40% is obtained when using HPCall as compared to the native 454 base-caller. The decrease is observed both for indels and for SNPs.

### Computational performance

The performance of HPCall was tested using a representative 454 dataset containing 198,347 sequences. Both the native 454 basecaller and Pyrobayes processed the dataset in approximately 4h, while the base-calling by HPCall was conducted in approximately 6.5h. Both Pyrobayes and HPCall had comparable memory footprints of less than 1GB. HPCall used 3.5GB hard disk space to store the preprocessed data before actual basecalling took place. The computational performance was measured on a 2×6 Core Intel Xeon X7460, 2.66 GHz Processors GNU/Linux server system with 128 GB RAM.

### Software package

The HPCall pipeline contains three modules. The first is a preprocessing module that stores all required data in a SQL database. The second module performs the actual base-calling by means of the R package . All base-calls, HPCall probabilities and quality scores are postprocessed in the final module to produce the final output files. The HPCall software and manual are available at https://sourceforge.net/projects/hpcall/.

## Discussion

One of the main contributions of HPCall is that the base-calling and quality score assignment are seamlessly integrated and occur simultaneously, instead of in two separate steps. For a given cycle and nucleotide, the probability of being the correct HPL is estimated for each possible HPL based on different noise predictors, and the call corresponds to the HPL with the maximum probability. In this way the extent of the maximal probability provides direct information about the base-calling uncertainty and can thus be used as a measure for the base-calling quality. Moreover, in the case of a miscall the second largest probability indicates whether an undercall or an overcall is more likely. This information is important for the downstream analysis of sequencing data, but it is completely lacking from traditional *Phred*-like quality scores produced by current 454 base-callers. The distributions of maximum base-calling probabilities in case of a miscall are more evenly distributed between 0.5 and 1 than in the case of a correct call where it is very often nearly 1. This suggests that relatively small maximum probabilities are often associated with miscalls and therefore should raise caution.

Because they are commonly used in the analysis of NGS experiments, HPCall also calculates *Phred*-like quality scores, based on the base-calling probabilities. These can be used in the same way as 454 quality scores. They are related to the probability of not having an overcall. These ‘overcall’ quality scores appear to compete well with the 454 quality scores, while the Pyrobayes quality scores perform clearly worse. At the same time, however, HPCall produces considerably more high-quality scores. Since we have all possible base-calling probabilities at our disposal, we can also calculate alternative quality scores based on the probability of not having an undercall. Subsequently, a summarizing *Phred*-like quality score is constructed by determining which of these two quality scores has the smallest value at the base-called HPL and this information is coded by the sign of the quality score (minus for undercall, plus for overcall). This new quality score now also contains information about the direction of a possible miscall. Quality-aware sequence aligners may use these scores to provide more reliable mapping results. We further illustrate the use of the HPCall base-calling probabilities and the *Phred*-like HPCall quality scores for assessing indels in sequence variant detection. In each sequencing flow, the native 454 base-caller produces quality scores for each called base, i.e. for a homopolymer of length 3, also 3 quality scores are provided. These quality scores are not informative to discriminate between potential undercalls or overcalls. Furthermore, in the situation that 0 bases are called instead of 1, no quality scores are provided by the other base-callers. Hence, no information is given about the probability that indeed 0 bases should have been called. In contrast, HPCall clearly indicates which type of miscall - undercall or overcall - is possibly to be expected in these examples, by means of the second highest base-calling probability and the sign of the HPCall quality score.

Besides the added value of the base-calling probabilities and quality scores, the prediction accuracy of HPCall surpasses that of the native 454 base-caller and of Pyrobayes. Based on our *E. coli* data we detect a 35% reduction of base-calling errors as compared to the current 454 base-caller. This reduction is quite stable throughout the whole HPL range. This number is based on a model that uses not only information from the preprocessed flowgram values, but also from the earlier-stage raw intensities to call the HPL in each flow of the sequencing process. If only information of flowgram values is used, the reduction of base-calling errors is still there, but it is smaller. Hence, although preprocessing raw intensities to flowgram values in a separate step prior to base-calling has the merit of reducing the spatial, read-specific and background optical noise in the data to a large extent, it also seems to remove crucial information for the base-calling task itself. The lower number of base-calling errors is also reflected in the lower number of detected indels and SNPs after mapping the base-called reads to the *E. coli* reference sequence. The beneficial performance of HPCall was confirmed on a 454 amplicon resequencing experiment of the human TP53 gene. When HPCall is run using the model trained on the *E. coli* data set the base-calling accuracy slightly decreases (see Additional file 12). For optimal results, it is therefore recommended to retrain the model for different experiments. For calibration of the base-caller the associated HPLs of a reference sequence are used to fit the model. A possible way to implement this is by adding plasmids to the sequencing experiment. The 454 sequencer uses control reads containing varying HPLs for recalibrating its native base-caller. Hence, these control reads would be very valuable for this purpose. Up to now, however, the 454 software does not allow to extract the flowgram values associated with these reads. The larger accuracy and creation of the more informative quality scores by HPCall comes at the cost of an additional computing time that is in the same order as the time for native 454 base-calling. Based on the *E. coli* data, the accuracy performance of HPCall is stable across different training data sets used to fit the model.

While HPCall was primarily developed for base-calling of 454 data, it has the potential to be used for other homopolymer-sensitive sequencing platforms as well, e.g. the PGM sequencer of Ion Torrent. Within the broad framework of the Hurdle Poisson model the algorithms to train the model remain unchanged. This also means that similar informative quality scores can be produced for other platforms. Only the explanatory variables used to predict the HPL will be specific for each platform, e.g. the nucleotide flow order of the Ion PGM sequencer is different from the 454 sequencer. Although it was not the main focus of this research, a first test with PGM 314 *E. coli* reference data already shows promising results (details in Additional file 12).

## Conclusions

In this paper, we have proposed an alternative method for the base-calling of 454 pyrosequencing data, referred to as HPCall. Based on the obtained results, we strongly believe that using HPCall for base-calling and taking advantage of the base-calling probabilities in downstream tasks like mapping, genome assembly and sequence variant detection will lead to more accurate and powerful applications. Although HPCall is developed based on sequencing data of the 454 sequencing system, the underlying probabilistic framework is quite general. Therefore, we expect it to be rather straightforward to adapt HPCall for use in novel emerging sequencing platforms based on flow cycles, for which base-calling of long homopolymers is critical, e.g. Ion Torrent PGM.

## Competing interests

The authors declare that they have no competing interests.

## Authors’ contributions

KDB, LC and OT developed the statistical method. KDB and LC implemented the method in R. KDB performed all the analyses and wrote the manuscript. JDS implemented the base-calling pipeline in Perl. WVC provided the data set. RI contributed to the method development and design of the paper. All authors read and approved the final manuscript.

## Supplementary Material

Additional file 1 **Probability of miscalls by native 454 base-caller.** Probability of miscalls by the native 454 base-caller for different HPLs. The base-calling error rate clearly increases by increasing HPL and becomes quite substantial from HPL 4. Click here for file

Additional file 2 **Overview of HPCall base-calling pipeline.** Overview of the HPCall base-calling pipeline. Different source files are merged in a data preparation step before the base-calling takes place. The output of the pipeline contains base-called sequence reads, *Phred*-like quality scores, and base-calling probabilities for the different HPLs. Click here for file

Additional file 3 **Smooth functions in Hurdle Poisson model.** Specification of the smooth functions *f*_*j*_ and *g*_*j*_ in the Hurdle Poisson model. Click here for file

Additional file 4 **Distribution of quality scores at HPLref3 undercall.** The empirical cumulative distribution function of 454 quality scores (upper) and HPCall quality scores *QS*_undercall_ (lower) for sequences with reference HPLref 3 assigned to bases associated with HPL 2, 3 and 4. Because of the undercall only 454 quality scores associated with HPL 2 are available. The HPCall quality scores associated with HPL 3 and HPL 4 are mostly very high, whereas this is not the case for those associated with HPL 2. HPCall clearly indicates that undercalls are more likely in this situation, whereas this insight is not provided by the 454 quality scores. Click here for file

Additional file 5 **Distribution of new informative quality scores at HPLref3.** The empirical cumulative distribution function of HPCall quality scores *QS*_HPCall_ for sequences with reference HPLref 3 assigned to bases associated with HPL 2, 3 and 4, in the case of an undercall (upper), correct call (middle) or overcall (lower). Click here for file

Additional file 6 **Histograms of estimated probabilities by HPCall.** (A) Histograms of the maximal estimated probabilities by HPCall in the case of a correct call (upper left), and (B) in the case of a miscall (upper right). (C) The histogram in the lower left panel gives the distribution of estimated probabilities for the reference HPLs in the case of a miscall. These very often correspond with the reference HPL. (D) The lower right panel gives the histogram of the sum of the probabilities given in the upper right and lower left panel. These two probabilities almost always sum to a value close to 1. Click here for file

Additional file 7 **Comparison of absolute number of base-calling errors.** Comparison of the absolute number of base-calling errors by HPL for the three base-calling methods. Using HPCall leads to an overall decrease of the number of base-calling errors of 35% compared to the native 454 base-caller. The lower number of base-calling errors for HPCall is consistent throughout the complete range of reference HPLs. Click here for file

Additional file 8 **Comparison of base-calling prediction accuracy.** Prediction accuracy for the different base-calling methods separated by nucleotide type. Although the prediction accuracy of the native base-caller is already quite high, HPCall obtains higher prediction accuracies for each individual nucleotide type. This is still the case if only flowgram values (fg) are used. Both HPCall and the native 454 base-caller clearly outperform Pyrobayes. Click here for file

Additional file 9 **Variability of the prediction accuracy by HPCall.** Variability of the prediction accuracy of HPCall. The obtained prediction accuracies are very stable among the different random samples of training data. The standard deviations of the prediction accuracies range from 0.000024 (for nucleotide C) to 0.000047 (for nucleotide T). Click here for file

Additional file 10 **Comparison of mapping mismatches.** Percentage of reads with different numbers of mismatches in the mapping between the reads produced by either HPCall or the native 454 base-caller and the *E. coli* K-12 reference sequence. For mapping either *ssaha2* or *subread* is used. Detected number of sequence variants for the *E. coli* data set using *ssahaSNP*. HPCall results in more perfect-matching reads and less overall indels and SNPs. Click here for file

Additional file 11 **Base-calling of human TP53 454 amplicon resequencing data.** Percentage of reads with different number of mismatches in the mapping between either HPCall (with or without training) or the native 454 base-caller and the human TP53 gene reference sequence. HPCall results in more perfect-matching reads. When trained on the *E. coli* data set the percentage of perfect-matching reads decreases slightly. Click here for file

Additional file 12 **Base-calling of PGM 314*E. coli*data with HPCall.** Cumulative percentage of reads as a function of mismatches per read in the mapping between the reads produced by either HPCall or the standard Ion PGM base-caller and the *E. coli* DH10B reference sequence. The results for HPCall are promising. Click here for file
