# Supplementary material for: Improved base-calling and quality scores for 454 sequencing based on a Hurdle Poisson model
Source: BMC Bioinformatics. 2012 Nov 15;13:303. doi: 10.1186/1471-2105-13-303 (PMC3534400; doi:10.1186/1471-2105-13-303)
Supplement: Additional file 1 — Probability of miscalls by native 454 base-caller. Probability of miscalls by the native 454 base-caller for different HPLs. The base-calling error rate clearly increases by increasing HPL and becomes quite substantial from HPL 4. [file 1471-2105-13-303-S1.pdf]

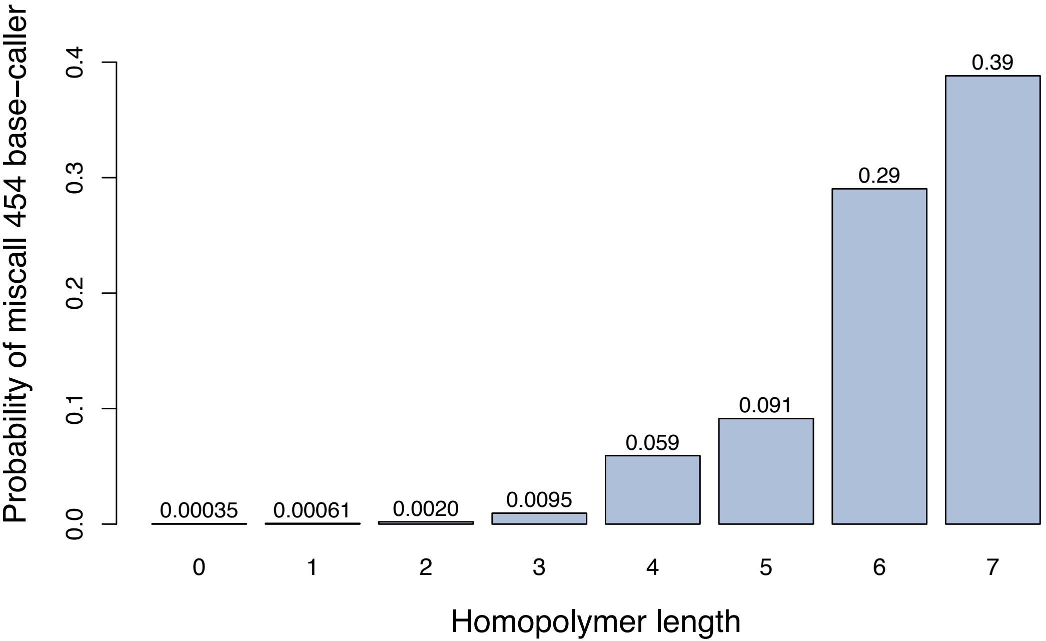

Figure 1: Probability of miscalls by the native 454 base-caller for different HPLs. The base-calling error rate clearly increases by increasing HPL and becomes quite substantial from HPL 4.
