# Supplementary material for: Improved base-calling and quality scores for 454 sequencing based on a Hurdle Poisson model
Source: BMC Bioinformatics. 2012 Nov 15;13:303. doi: 10.1186/1471-2105-13-303 (PMC3534400; doi:10.1186/1471-2105-13-303)
Supplement: Additional file 2 — Overview of HPCall base-calling pipeline. Overview of the HPCall base-calling pipeline. Different source files are merged in a data preparation step before the base-calling takes place. The output of the pipeline contains base-called sequence reads, Phred-like quality scores, and base-calling probabilities for the different HPLs. [file 1471-2105-13-303-S2.pdf]

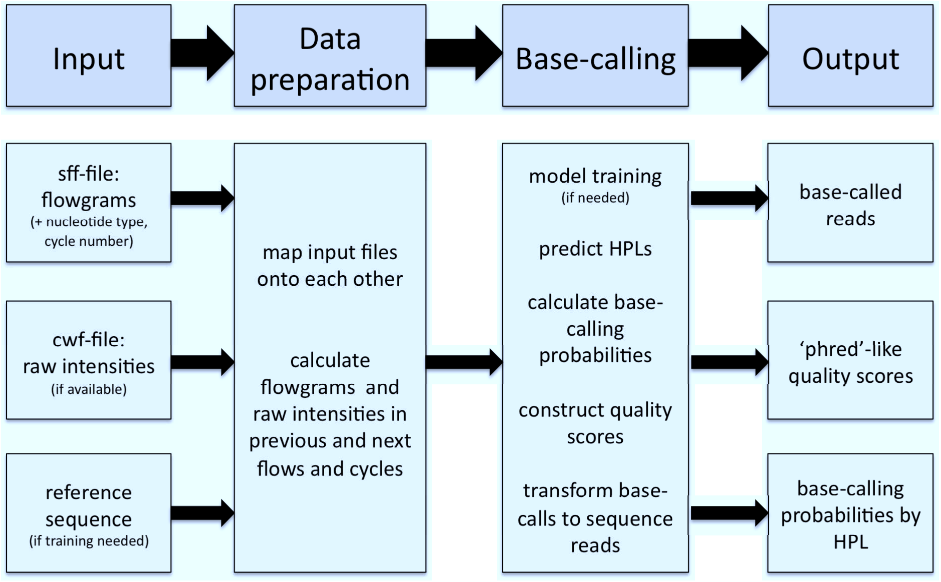

Figure 2: Overview of the HPCall base-calling pipeline. Different source files are merged in a data preparation step before the base-calling takes place. The output of the pipeline contains base-called sequence reads, *Phred*-like quality scores, and base-calling probabilities for the different HPLs.

Before running the Hurdle Poisson base-calling model a preliminary data preparation step is performed in HPCall. In this step several raw data files are merged to create a data set in flow space that can be passed to our base-calling algorithm. Both the flowgram values (*.sff*) and the raw intensities measured prior to signal processing (*.cwf*) can be used. It is also possible to only include information on the flowgram values if the raw intensities are no longer available. For calibration of the model the reference sequence data is first transformed from nucleotide space to flow space according to the flow order (TACG). Next, the flowgram values and raw intensities are mapped onto these reference HPLs in the corresponding sequencing cycle, together with other relevant information such as incorporated nucleotide type and cycle number. Also flowgram values and raw intensities in previous and next flows and cycles are calculated to be used in the base-calling model (see further). After the base-calling three output files are created: (a) the base-called reads, (b) the associated *Phred*-like quality scores, and (c) a file with the base-calling probabilities by HPL.
