# Supplementary material for: Improved base-calling and quality scores for 454 sequencing based on a Hurdle Poisson model
Source: BMC Bioinformatics. 2012 Nov 15;13:303. doi: 10.1186/1471-2105-13-303 (PMC3534400; doi:10.1186/1471-2105-13-303)
Supplement: Additional file 3 — Smooth functions in Hurdle Poisson model. Specification of the smooth functions fj and gj in the Hurdle Poisson model. [file 1471-2105-13-303-S3.pdf]

Smooth functions in Hurdle Poisson model

Consider the binomial and the the Poisson component of the Hurdle Poisson model which are both modeled with several predictors $x_{j,bc}$ and $y_{j,bc}$

$$\text{logit}(\pi_{bc}) = \beta_{0,bc} + \sum_{j=1}^{k} f_j(x_{j,bc})$$

$$\log(\lambda_{bc}) = \gamma_{0,bc} + \sum_{j=1}^{l} g_j(y_{j,bc})$$

In this model f_j and g_j are specified using cubic smoothing splines, which can be represented as a linear combination of a set of basis functions with m knots,

$$h(z) = \sum_{r=0}^{3} \delta_{0r} z^r + \sum_{s=1}^{m} \delta_s (z - t_s)_+^3,$$

where t_s, $s = 1, \ldots, m$ are the m knots (e.g. Thilakarathne et al., 2011). The different predictors used in the model are given in the main text.

Reference: Thilakarathne, P., Clement, L., Lin, D., Shkedy, Z., Kasim, A., Talloen, W., Versele, M., and Verbeke, G. (2011). The Use of Semi-parametric Mixed Models to Analyze PamChip Peptide Array Data: an Application to an Oncology Experiment. Bioinformatics, 27 (20), 2859-2865.
