# Supplementary material for: Improved base-calling and quality scores for 454 sequencing based on a Hurdle Poisson model
Source: BMC Bioinformatics. 2012 Nov 15;13:303. doi: 10.1186/1471-2105-13-303 (PMC3534400; doi:10.1186/1471-2105-13-303)
Supplement: Additional file 4 — Distribution of quality scores at HPLref3 undercall. The empirical cumulative distribution function of 454 quality scores (upper) and HPCall quality scores QSundercall (lower) for sequences with reference HPLref 3 assigned to bases associated with HPL 2, 3 and 4. Because of the undercall only 454 quality scores associated with HPL 2 are available. The HPCall quality scores associated with HPL 3 and HPL 4 are mostly very high, whereas this is not the case for those associated with HPL 2. HPCall clearly indicates that undercalls are more likely in this situation, whereas this insight is not provided by the 454 quality scores. [file 1471-2105-13-303-S4.pdf]

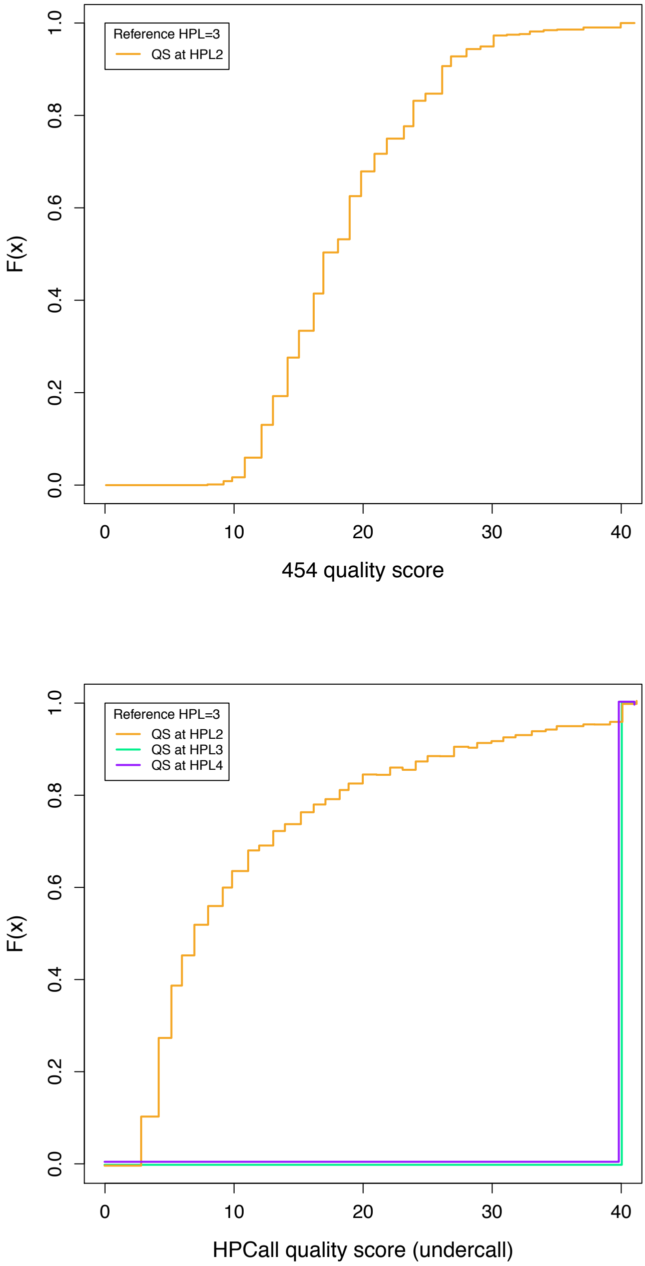

Figure 3: The empirical cumulative distribution function of 454 quality scores (upper) and HPCall quality scores $QS_{\text{undercall}}$ (lower) for sequences with reference HPLref 3 assigned to bases associated with HPL 2, 3 and 4. Because of the undercall only 454 quality scores associated with HPL 2 are available. The HPCall quality scores associated with HPL 3 and HPL 4 are mostly very high, whereas this is not the case for those associated with HPL 2. HPCall clearly indicates that undercalls are more likely in this situation, whereas this insight is not provided by the 454 quality scores.
