# Supplementary material for: Improved base-calling and quality scores for 454 sequencing based on a Hurdle Poisson model
Source: BMC Bioinformatics. 2012 Nov 15;13:303. doi: 10.1186/1471-2105-13-303 (PMC3534400; doi:10.1186/1471-2105-13-303)
Supplement: Additional file 5 — Distribution of new informative quality scores at HPLref3. The empirical cumulative distribution function of HPCall quality scores QSHPCall for sequences with reference HPLref 3 assigned to bases associated with HPL 2, 3 and 4, in the case of an undercall (upper), correct call (middle) or overcall (lower). [file 1471-2105-13-303-S5.pdf]

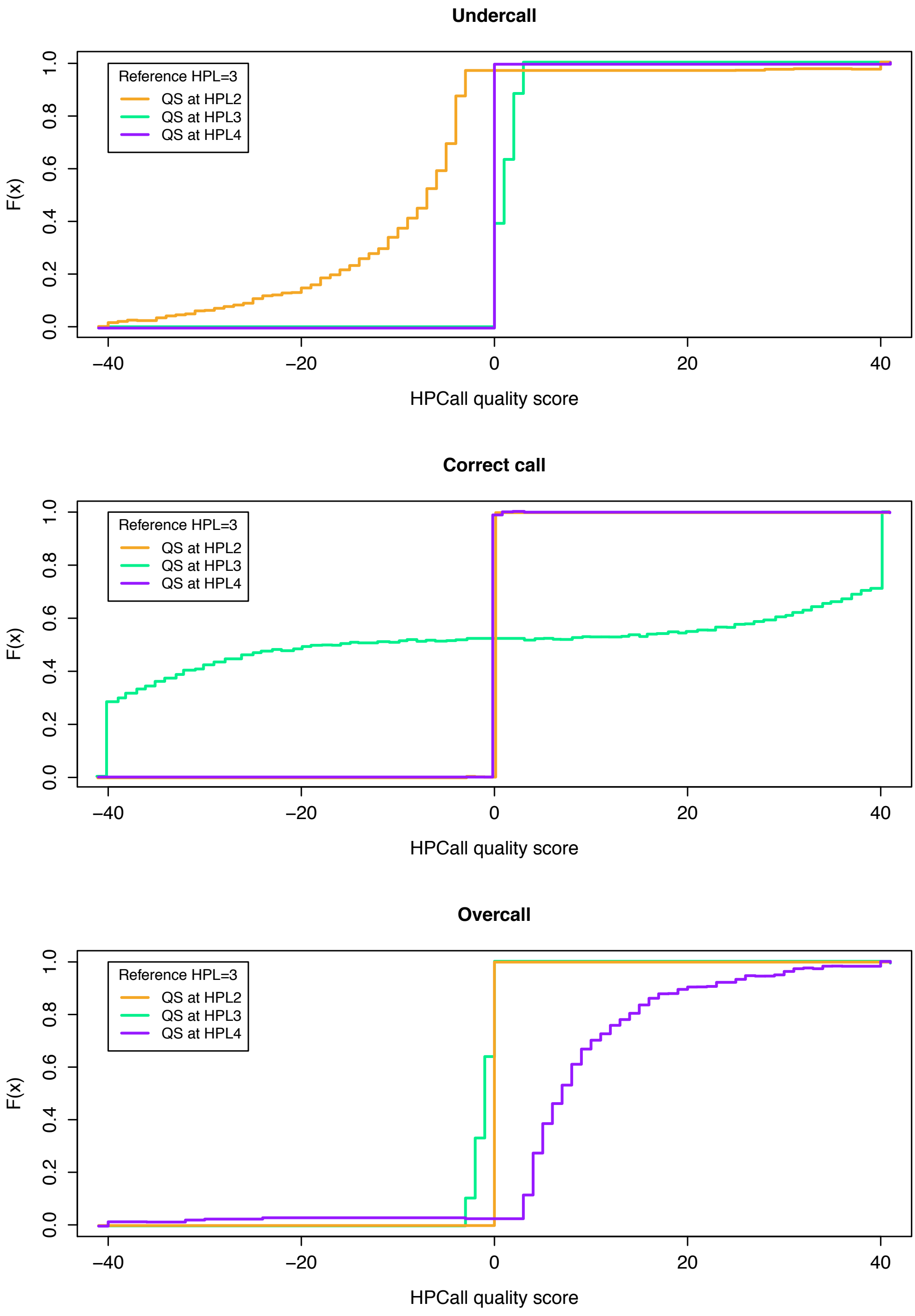

Figure 4: The empirical cumulative distribution function of HPCall quality scores QS_{HPCall} for sequences with reference HPLref 3 assigned to bases associated with HPL 2, 3 and 4, in the case of an undercall (upper), correct call (middle) or overcall (lower).
