# Supplementary material for: Improved base-calling and quality scores for 454 sequencing based on a Hurdle Poisson model
Source: BMC Bioinformatics. 2012 Nov 15;13:303. doi: 10.1186/1471-2105-13-303 (PMC3534400; doi:10.1186/1471-2105-13-303)
Supplement: Additional file 6 — Histograms of estimated probabilities by HPCall. (A) Histograms of the maximal estimated probabilities by HPCall in the case of a correct call (upper left), and (B) in the case of a miscall (upper right). (C) The histogram in the lower left panel gives the distribution of estimated probabilities for the reference HPLs in the case of a miscall. These very often correspond with the reference HPL. (D) The lower right panel gives the histogram of the sum of the probabilities given in the upper right and lower left panel. These two probabilities almost always sum to a value close to 1. [file 1471-2105-13-303-S6.pdf]

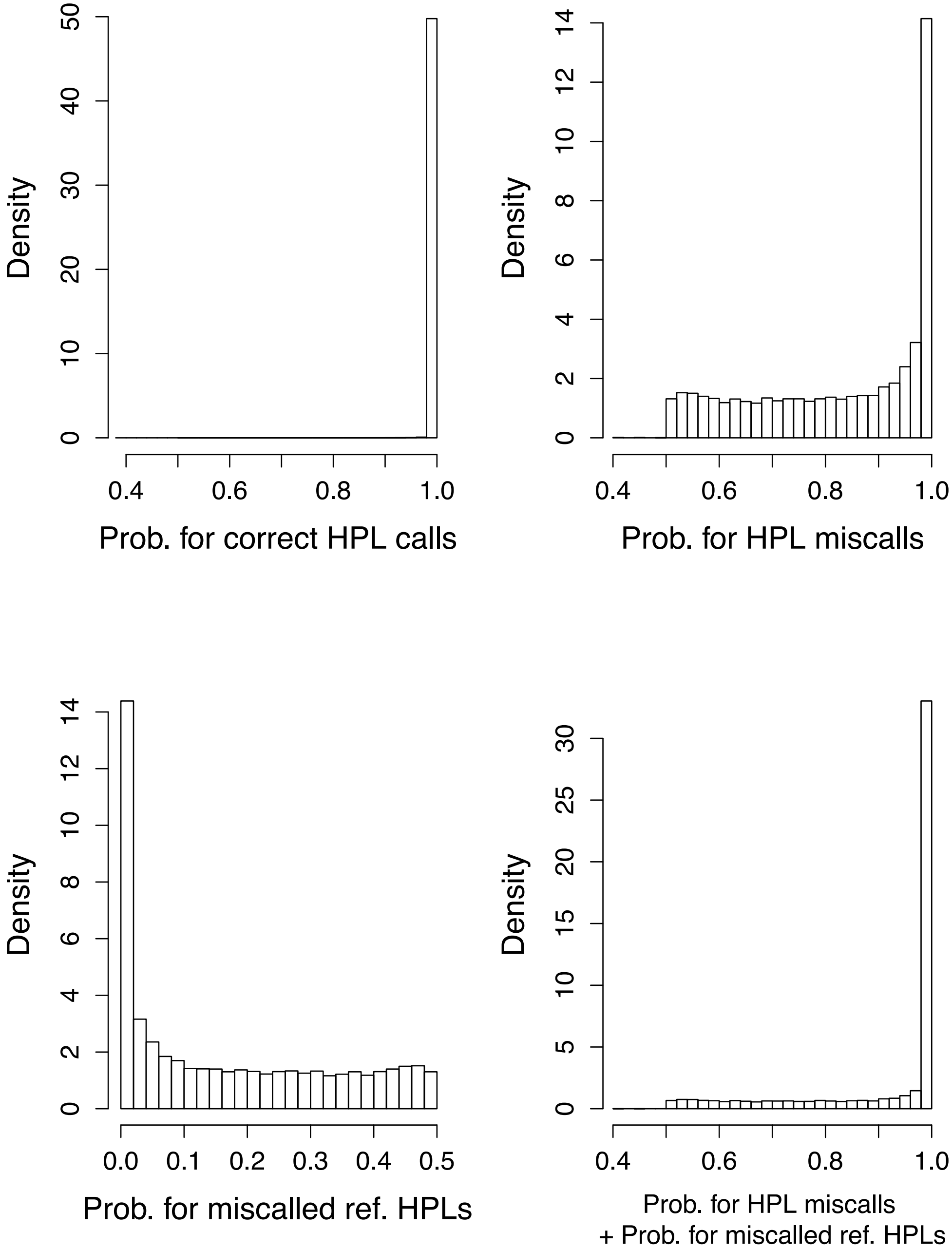

Figure 5: (A) Histograms of the maximal estimated probabilities by HPCall in the case of a correct call (upper left), and (B) in the case of a miscall (upper right). (C) The histogram in the lower left panel gives the distribution of estimated probabilities for the reference HPLs in the case of a miscall. These very often correspond with the reference HPL. (D) The lower right panel gives the histogram of the sum of the probabilities given in the upper right and lower left panel. These two probabilities almost always sum to a value close to 1.
