# Supplementary material for: Improved base-calling and quality scores for 454 sequencing based on a Hurdle Poisson model
Source: BMC Bioinformatics. 2012 Nov 15;13:303. doi: 10.1186/1471-2105-13-303 (PMC3534400; doi:10.1186/1471-2105-13-303)
Supplement: Additional file 7 — Comparison of absolute number of base-calling errors. Comparison of the absolute number of base-calling errors by HPL for the three base-calling methods. Using HPCall leads to an overall decrease of the number of base-calling errors of 35% compared to the native 454 base-caller. The lower number of base-calling errors for HPCall is consistent throughout the complete range of reference HPLs. [file 1471-2105-13-303-S7.pdf]

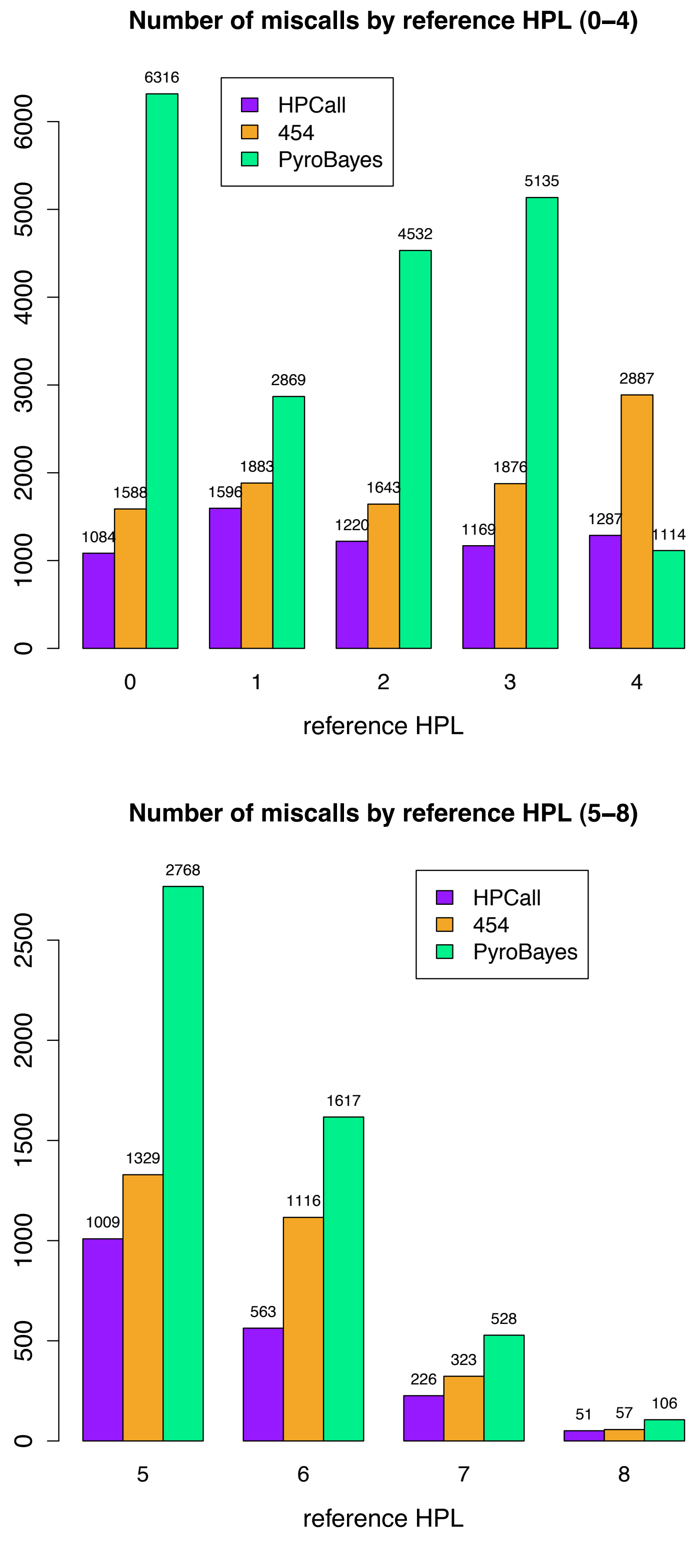

Figure 6: Comparison of the absolute number of base-calling errors by HPL for the three base-calling methods. Using HPCall leads to an overall decrease of the number of base-calling errors of 35% compared to the native 454 base-caller. The lower number of base-calling errors for HPCall is consistent throughout the complete range of reference HPLs.
