# Supplementary material for: Improved base-calling and quality scores for 454 sequencing based on a Hurdle Poisson model
Source: BMC Bioinformatics. 2012 Nov 15;13:303. doi: 10.1186/1471-2105-13-303 (PMC3534400; doi:10.1186/1471-2105-13-303)
Supplement: Additional file 8 — Comparison of base-calling prediction accuracy. Prediction accuracy for the different base-calling methods separated by nucleotide type. Although the prediction accuracy of the native base-caller is already quite high, HPCall obtains higher prediction accuracies for each individual nucleotide type. This is still the case if only flowgram values (fg) are used. Both HPCall and the native 454 base-caller clearly outperform Pyrobayes. [file 1471-2105-13-303-S8.pdf]

Table 1: Prediction accuracy for the different base-calling methods separated by nucleotide type. Although the prediction accuracy of the native base-caller is already quite high, HPCall obtains higher prediction accuracies for each individual nucleotide type. This is still the case if only flowgram values (fg) are used. Both HPCall and the native 454 base-caller clearly outperform Pyrobayes.

	correct HPLs (%)				
	A	C	G	T	Overall
HPCall	99.86	99.95	99.94	99.88	99.91
HPCall (only fg)	99.80	99.94	99.94	99.86	99.89
native 454	99.72	99.94	99.93	99.84	99.86
Pyrobayes	99.50	99.85	99.84	99.67	99.72
