# Supplementary material for: Improved base-calling and quality scores for 454 sequencing based on a Hurdle Poisson model
Source: BMC Bioinformatics. 2012 Nov 15;13:303. doi: 10.1186/1471-2105-13-303 (PMC3534400; doi:10.1186/1471-2105-13-303)
Supplement: Additional file 9 — Variability of the prediction accuracy by HPCall. Variability of the prediction accuracy of HPCall. The obtained prediction accuracies are very stable among the different random samples of training data. The standard deviations of the prediction accuracies range from 0.000024 (for nucleotide C) to 0.000047 (for nucleotide T). [file 1471-2105-13-303-S9.pdf]

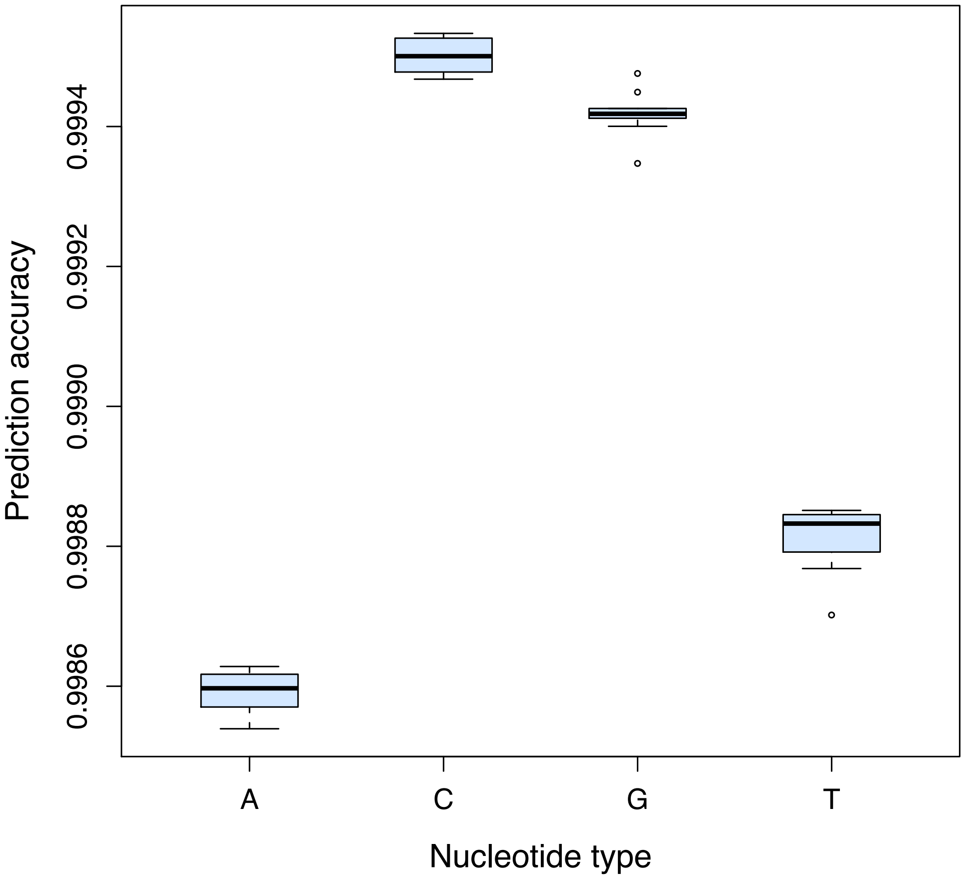

Figure 7: Variability of the prediction accuracy of HPCall. The obtained prediction accuracies are very stable among the different random samples of training data. The standard deviations of the prediction accuracies range from 0.000024 (for nucleotide C) to 0.000047 (for nucleotide T).
