# Supplementary material for: Improved base-calling and quality scores for 454 sequencing based on a Hurdle Poisson model
Source: BMC Bioinformatics. 2012 Nov 15;13:303. doi: 10.1186/1471-2105-13-303 (PMC3534400; doi:10.1186/1471-2105-13-303)
Supplement: Additional file 10 — Comparison of mapping mismatches. Percentage of reads with different numbers of mismatches in the mapping between the reads produced by either HPCall or the native 454 base-caller and the E. coli K-12 reference sequence. For mapping either ssaha2 or subread is used. Detected number of sequence variants for the E. coli data set using ssahaSNP. HPCall results in more perfect-matching reads and less overall indels and SNPs. [file 1471-2105-13-303-S10.pdf]

Table 2: Percentage of reads with different numbers of mismatches in the mapping between the reads produced by either HPCall or the native 454 base-caller and the *E. coli* K-12 reference sequence. For mapping either *ssaha2* or *subread* is used. HPCall results in more perfect-matching reads.

Mapping	*ssaha2*		*subread*	
	HPCall	native 454	HPCall	native 454
Number of errors per read (%)				
0	66.03	56.37	69.42	60.42
1	22.97	26.78	22.25	26.08
2	6.81	10.20	5.53	8.53
3	2.45	3.79	1.81	3.11
4	0.90	1.54	0.60	1.12
5	0.84	1.32	0.40	0.74

Table 3: Detected number of sequence variants for the *E. coli* data set using *ssahaSNP*. HPCall results in less overall indels and SNPs.

SNP calling	*ssahaSNP*	
	HPCall	native 454
Indels	4954	8388
SNPs	528	756
Total sequence variants	5482	9144
