# Supplementary material for: Improved base-calling and quality scores for 454 sequencing based on a Hurdle Poisson model
Source: BMC Bioinformatics. 2012 Nov 15;13:303. doi: 10.1186/1471-2105-13-303 (PMC3534400; doi:10.1186/1471-2105-13-303)
Supplement: Additional file 11 — Base-calling of human TP53 454 amplicon resequencing data. Percentage of reads with different number of mismatches in the mapping between either HPCall (with or without training) or the native 454 base-caller and the human TP53 gene reference sequence. HPCall results in more perfect-matching reads. When trained on the E. coli data set the percentage of perfect-matching reads decreases slightly. [file 1471-2105-13-303-S11.pdf]

Base-calling of human TP53 454 amplicon resequencing data

Amplicon DNA templates of exons 4 to 7 (forward and reverse strand) in the human TP53 gene were sequenced using the Roche/454 sequencing platform. The TP53 gene encodes for a protein called tumor protein p53. This protein plays an important role in the cell cycle. By activating expression of downstream genes that inhibit growth and/or invasion, it can function as a tumor suppressor. The 90382 reads are base-called by both the native 454 base-caller and by HPCall and mapped to the TP53 reference genome using ssaha2. Table 4 shows the percentage of different numbers of mismatches, indicating that also for this data set HPCall results in more perfect-matching reads. The percentage of perfect-matching reads decreases slightly when the model is trained on the E. coli data set.

Table 4: Percentage of reads with different numbers of mismatches in the mapping between the reads produced by either HPCall or the native 454 base-caller and the human TP53 gene reference sequence. HPCall results in more perfect-matching reads. When trained on the *E. coli* data set, the performance decreases slightly.

	native 454	HPCall	HPCall trained on *E. coli*
Number of errors per read (%)			
0	74.96	77.05	72.25
1	19.14	18.69	18.19
2	3.72	2.65	5.53
3	1.16	0.85	2.26
4	0.60	0.34	0.76
5 or more	0.42	0.42	1.01
