# Supplementary material for: Improved base-calling and quality scores for 454 sequencing based on a Hurdle Poisson model
Source: BMC Bioinformatics. 2012 Nov 15;13:303. doi: 10.1186/1471-2105-13-303 (PMC3534400; doi:10.1186/1471-2105-13-303)
Supplement: Additional file 12 — Base-calling of PGM 314E. colidata with HPCall. Cumulative percentage of reads as a function of mismatches per read in the mapping between the reads produced by either HPCall or the standard Ion PGM base-caller and the E. coli DH10B reference sequence. The results for HPCall are promising. [file 1471-2105-13-303-S12.pdf]

Base-calling of PGM 314 *E. coli* data with HPCall

The applicability of HPCall for the PGM sequencer of Ion Torrent is tested on a PGM 314 *E. coli* DH10B data set (retrieved from http://ioncommunity.lifetechnologies.com/docs/DOC-1848) by using the flow values provided in the SFF file. The base-called reads from HPCall and the standard PGM software are mapped to the *E. coli* DH10B reference genome using ssaha2. Figure 8 gives the cumulative percentage of reads as a function of number of mismatches per read. This figure indicates that HPCall is a promising alternative for the base-calling of Ion PGM sequencing data, as it results in more base-called reads with a low number of mismatches.

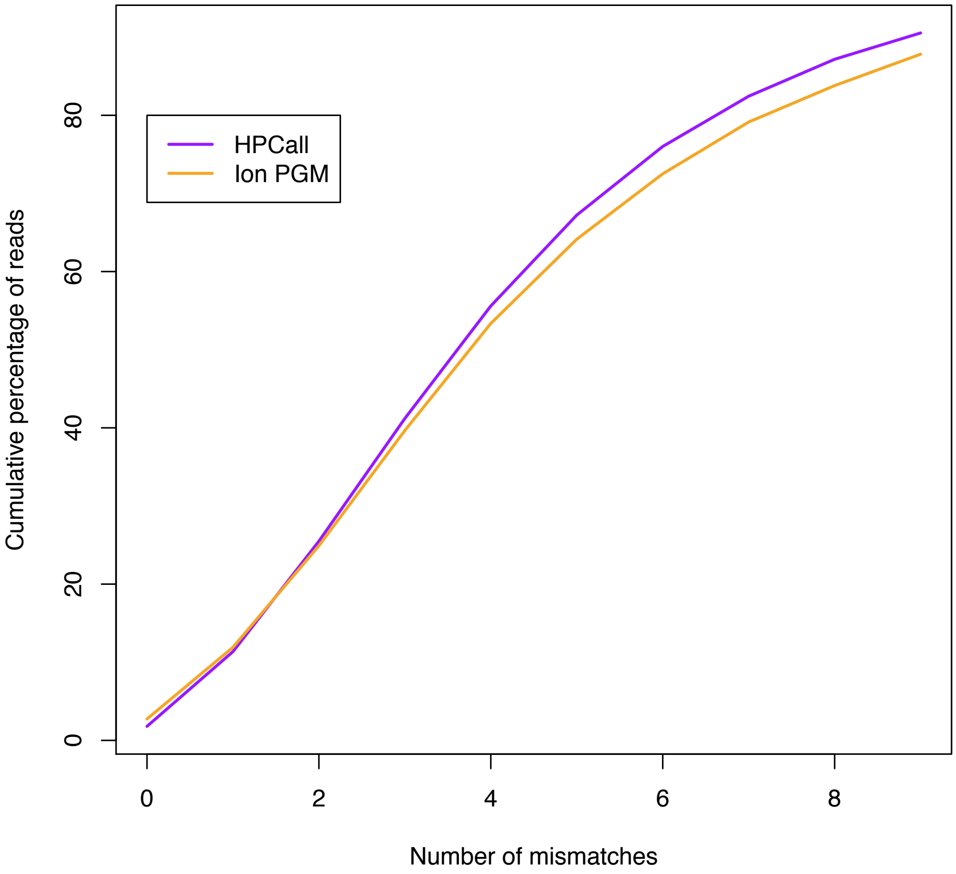

Figure 8: Cumulative percentage of reads as a function of mismatches per read in the mapping between the reads produced by either HPCall or the standard Ion PGM base-caller and the *E. coli* DH10B reference sequence. The results for HPCall are promising.
